# Correlation between anxiety-depression status and cytokines in diarrhea-predominant irritable bowel syndrome

**DOI:** 10.3892/etm.2013.1101

**Published:** 2013-05-08

**Authors:** JINGGUO GAO

**Affiliations:** Department of Digestive System, Xingtai People’s Hospital, Xingtai, Hebei 054031, P.R. China

**Keywords:** irritable bowel syndrome, cytokine, IL-1β, IL-10, anxiety, depression

## Abstract

The aim of this study was to investigate the correlation between anxiety-depression status and cytokines in diarrhea-predominant irritable bowel syndrome (IBS-D). IBS-D patients were divided into an anxiety-depression IBS-D group and a non-anxiety-depression IBS-D group. Patients without IBS, anxiety or depression were selected as the control group. Scoring was performed using the self-rating anxiety scale (SAS) and self-rating depression scale (SDS). Levels of IL-1β and IL-10 in the blood and sigmoid colon mucosa were detected, and the proportions of IL-1β- and IL-10-positive cells in the sigmoid colon mucosa were determined. The results demonstrated that the SDS and SAS scores in the IBS-D group were significantly higher than those in the control group (P<0.05). The levels of IL-1β in the blood and sigmoid colon mucosa and the proportion of IL-1β-positive cells in the sigmoid colon mucosa in the IBS-D group were significantly higher than those in the control group (P<0.05). The levels of IL-10 in the blood and sigmoid colon mucosa and the proportion of IL-10-positive cells in the IBS-D group were significantly lower than those in the control group (P<0.05). The levels of IL-1β in the blood and sigmoid colon mucosa and the proportion of IL-1β-positive cells in the anxiety-depression IBS-D group were significantly higher than those in the non-anxiety-depression IBS-D group, and the levels of IL-10 and the proportion of IL-10-positive cells in the anxiety-depression IBS-D group were significantly lower than those in the non-anxiety-depression IBS-D group (P<0.05). Anxiety-depression status may cause the IL-1β and IL-10 levels in IBS patients to change and result in an imbalance of the proinflammatory and anti-inflammatory cytokines, leading to the occurrence or aggravation of IBS.

## Introduction

Irritable bowel syndrome (IBS) refers to a group of symptoms that are persistent or intermittent but are not associated with clear morphological changes or biochemical abnormalities. IBS manifests as abdominal pain and abdominal distension, as well as abnormalities in bowel evacuation habit and stool characteristics for unknown reasons (1). IBS is a heterogeneous disease caused by numerous factors that are interelated with each other. Among these factors, psychology, emotional state, society and environment play important roles in the development of IBS. Furthermore, nervous, immune and endocrine systems also contribute to the development of this medical condition, leading to intestinal smooth muscle movement disorders and visceral paresthesia (2). The view that an inflammatory reaction exists in IBS patients has attracted extensive attention (3). The possible interaction between psychological abnormalities and the immune system has also been emphasized. In the current study, we explored the psychological status of IBS patients, as well as the mechanism of action of cytokines.

## Materials and methods

### General data

IBS patients who received treatment at Xingtai People’s Hospital (Hebei, China) between March 2008 and September 2010 were enrolled. This study was conducted in accordance with the Declaration of Helsinki and approved by the Ethics Committee of Xingtai People’s Hospital. Written informed consent was obtained from all participants. The participants were grouped based on the following standards: i) Rome III standards: recurrent abdominal pain or indisposition that started no less than six months before diagnosis and occurred on at least three days a month within the most recent three months. This phenomenon had to be accompanied by at least two out of the following three situations (not necessarily occurring in succession): a) improvement following defecation; b) disease accompanied by a change in defecation frequency (3/day or <3/week); and c) disease accompanied by changes in stool characteristics; ii) IBS typing standards based on the stool characteristic typing proposed by the Rome III team. Diarrhea-predominant IBS (IBS-D) is defined as pasty/watery stools >25% and massive/hard stools <25%.

Patients meeting the following standards were excluded: i) organic diseases, including ulcer, erosion and mass according to endoscopy; ii) organic disease in the liver, gall, pancreas or intestinal tract according to laboratory, ultrasonic and X-ray examination; iii) heart disease, diabetes, thyroid disease, connective tissue disease or psychosis; iv) administration of cathartics, antidiarrheals, prokinetic drugs or digestive enzyme drugs within one month; and v) history of abdominal surgery. Patients’ anxiety and depression were respectively evaluated using the self-rating anxiety scale (SAS) and self-rating depression scale (SDS). Patients meeting the diagnostic criteria of IBS-D were randomized into a normal psychological status IBS-D group and an anxiety-depression IBS-D group. The former was composed of 12 patients, including 6 males and 6 females. Their ages ranged from 17 to 61 years with an average age of 39 years. The latter consisted of 16 patients, including 6 males and 10 females. Their ages ranged from 17 to 58 years with an average age of 37.5 years. In addition, 15 patients with colonic polyps and internal hemorrhoids who received a health examination at the same hospital during the same period were selected as the control group (non-anxiety-depression non-IBS patients). The 15 patients of the control group contained 6 males and 9 females. Their ages ranged from 20 to 57 years with an average age of 38.5 years. The control subjects had no digestive symptoms, including abdominal pain, diarrhea and constipation, or nervous symptoms. Additionally, they had not recently been treated with any drugs.

### Specimens

Venous blood (2 ml) was extracted on an empty stomach in the morning for supernatant determination. Two mucous membranes from the sigmoid colon were obtained from each participant for the preparation of biopsy specimens. One was fixed in neutral formalin for interleukin (IL)-1β and IL-10 immunohistochemistry and the other was immediately frozen in liquid nitrogen for radioimmunoassay.

### Radioimmunoassay

The sigmoid colon mucosa specimen was placed in a homogenizer, 0.1 M acetic acid was added and the speciment was then fully homogenized. The homogenate liquid was transferred to a plastic tube and left to stand overnight. An equal volume of 0.1 M sodium hydroxide solution was added, followed by centrifuging at 1409 × g for 15 min. The supernatant was obtained for detection. The sigmoid colon mucosa and blood specimens were analyzed using a SN-695B radioimmunoassay intelligent measuring instrument (Shanghai Institute of Nuclear Research, Shanghai, China) using a sequential saturation sampling approach, and a standard curve was automatically drawn.

### Immunohistochemistry

Mucosal specimens were fixed in 10% neutral formalin, routinely dehydrated and embedded in paraffin, then serially sectioned. The obtained sections were dewaxed, hydrated and then incubated with 0.3% methanol-hydrogen peroxide for 10 min at room temperature to block endogenous peroxidase activity. The sections were rinsed with distilled water and saturated in phosphate-buffered saline (PBS; pH 6.0) for 5 min for antigen repair. The sections were incubated with IL-1β and IL-10 primary antibodies (1:700 and 1:50 in dilution, respectively) at 37°C for 1 h. PBS replaced the primary antibodies for the control. Following PBS washing, the sections were incubated with 50 *μ*l biotinylated secondary antibody working solution at 37°C for 30 min. After rinsing with PBS for 10 min, 100 *μ*l freshly-prepared diaminobenzidine solution (containing a drop of 3% H_2_O_2_) was added to each section for coloration. Then, the sections were stained with hematoxylin, dehydrated and mounted.

### Outcome evaluation

All the specimens were observed using a Motic Med 6.0 digital medical image analysis system (Shanghai Xinzhen Equipment Co., Ltd., Shanghai, China) under the same amplifying power. For each section, ten visual fields were selected. The average optical density (OD) of positively stained cells was used to represent the level of antigen expression. The expression was semiquantitated through computed image analysis. A higher than average OD value indicated a higher expression level of IL-1β and IL-10 in the cells.

### Statistical analysis

All data are presented as mean ± standard deviation and analyzed by SPSS 13.0 software (SPSS, Inc., Chicago, IL, USA. The t-test was performed for comparisons between groups. P<0.05 was considered to indicate a statistically significant difference.

## Results

### Comparison of anxiety and depression between the IBS and control groups

As shown in Table I, the SDS and SAS scores in the IBS-D group were 40.98±11.76 and 48.32±14.32, respectively, and those in control group were 34.12±10.3 and 37.65±11.97, respectively. The SDS and SAS scores in the IBS-D group were significantly higher than those in the control group (P<0.05).

### Comparison of IL-1β and IL-10 levels between the IBS and control groups

The data shown in Table II indicate that the levels of IL-1β in the blood and sigmoid colon mucosa in the IBS-D group (20.12±1.03 and 15.97±0.83, respectively) were significantly increased compared with those in the control group (14.39±0.94 and 10.44±1.82, respectively; P<0.05). However, the levels of IL-10 in the blood and sigmoid colon mucosa in IBS-D group (25.68±14.36 and 21.56±1.73, respectively) were significantly lower than those in the control group (37.56±9.34 and 30.16±2.18, respectively; P<0.05).

### Comparison of IL-1β and IL-10 levels between the IBS groups

As shown in Table III, the levels of IL-1β in the blood and sigmoid colon mucosa in the anxiety-depression IBS-D group (21.65±0.78 and 20.86±1.34, respectively) were significantly higher than those in the non-anxiety-depression IBS-D group (18.35±1.37 and 16.28±0.97, respectively; P<0.05). However, the levels of IL-10 in the blood and sigmoid colon mucosa in the anxiety-depression IBS-D group (27.36±8.24 and 24.31±5.66, respectively) were significantly lower than those in the non-anxiety-depression IBS-D group (30.34±7.33 and 28.45±3.34, respectively; P<0.05).

### Comparison of IL-1β- and IL-10-positive cells between the IBS and control groups

The results shown in Table IV show that the proportion of IL-1β-positive cells in the IBS-D group (0.298±0.312) was significantly higher than that in the control group (0.091±0.036; P<0.05). The proportion of IL-10-positive cells in the IBS-D group (0.083±0.045) was significantly lower than that in the control group (0.159±0.138; P<0.05).

### Comparison of IL-1β- and IL-10-positive cells between the IBS groups

As shown in Table V, the proportion of IL-1β-positive cells in the anxiety-depression IBS-D group (0.310±0.223) was significantly higher than that in the non-anxiety-depression IBS-D group (0.234±0.124; P<0.05). However, the proportion of IL-10-positive cells in the anxiety-depression IBS-D group (0.075±0.097) was significantly lower than that in the non-anxiety-depression IBS-D group (0.104±0.257; P<0.05).

## Discussion

IBS refers to a group of symptoms that are persistent or intermittent but without noticeable morphological changes or biochemical abnormalities, represented by abdominal pain and abdominal distension, as well as abnormalities in bowel evacuation habit and stool characteristics for unknown reasons (4,5). The fundamental mechanism underlying these symptoms is based on abnormalities in intestinal movement dysfunction due to intestinal hypersensitivity and hyper-reactivity (6). In the current study, the results demonstrated that the IBS patients had significantly increased anxiety and depression scores compared with the control group. This finding indicates that IBS patients are subjected to noticeable psychological abnormalities. Mental stress may lead to basic immune dysfunction. The effect of mental stress on gastrointestinal sensation and movement may involve the neuroendocrine-immune network (7–9). IL-1β is an important pro-inflammatory factor, which induces an inflammatory reaction and destroys the integrity of the mucosal barrier. It directly acts on colonic smooth muscle to result in constriction and inflammatory pain. It also suppresses the absorption of water and sodium, resulting in diarrhea. Furthermore, it promotes an increase in the sensitivity of gastrointestinal cholinergic nerves to acetylcholine and other inflammatory mediators (10–12). IL-10 is a powerful immunological suppressor factor with pleiotropic biological activity. The genetic susceptibility of certain IBS patients to inflammation may result from insufficient secretion of the inflammation-suppressing factor IL-10 and transforming growth factors (5,13,14). Depression not only manifests as mental symptoms, but also extensively interacts with the immune system. Mental anomalies may lead to immunoloregulatory dysfunction. Conversely, pro-inflammatory factors may induce mental anomalies. This suggests that IBS patients are subjected to complicated changes of their nervous, endocrine and immune systems (15–17).

In the current study, IBS-D patients that met the anxiety-depression criteria were selected according to the criteria of the anxiety-depression scales and then compared with the normal control and non-anxiety-depression IBS patients to determine differences in the levels of IL-1β and IL-10 in the blood and sigmoid mucous membranes, as well as mucosal positive cell expression. The results demonstrated that the IL-1β level in the colonic mucous membrane and blood of the IBS-D group were significantly increased compared with those of the control group, whereas the levels of IL-10 were markedly reduced. The levels of IL-1β and IL-10 in the sigmoid colon mucosal membranes and blood of the anxiety-depression IBS-D group were significantly different from those of the non-anxiety-depression IBS group. Furthermore, immunohistochemistry revealed significant differences in the positive expression of IL-1β and IL-10 between the anxiety-depression and non-anxiety-depression IBS-D groups. These findings suggest that psychological status affects the levels of cytokines and induces or aggravates the digestive symptoms in IBS-D patients. Furthermore, chronic somatic symptoms aggravate patients’ psychological burden, thereby leading to the aggravation of anxiety and depression. Thus, somatic symptoms and psychological status interact with or even promote each other (18–21). Cytokines, inflammatory reactions and psychological factors all play important roles in the development of IBS. Psychological factors lead to variations in the levels of cytokines and the balance between anti-inflammatory and pro-inflammatory factors, resulting in the aggravation of IBS or digestive symptoms; whereas the aggravation of somatic symptoms exacerbates psychological burden and anxiety-depression symptoms, exhibiting a close interactive correlation (22–24).

IBS is a heterogeneous disease caused by numerous factors. Anxiety-depression status may cause changes in the IL-1β and IL-10 levels of IBS patients resulting in an imbalance of the proinflammatory and anti-inflammatory cytokines, leading to the occurrence or aggravation of IBS.

## Figures and Tables

**Table I.** Comparison of the anxiety and depression scores between the IBS group and the control group.

Group	SDS	SAS
Control	34.12±10.3	37.65±11.97
IBS-D	40.98±11.76^a^	48.32±14.32^a^

a P<0.05 compared with the control group. IBS, irritable bowel syndrome; IBS-D, diarrhea-predominant IBS; SDS, self-rating depression scale; SAS, self-rating anxiety scale.

**Table II.** Comparison of IL-1β and IL-10 levels between the IBS group and the control group.

Group	IL-1β (pg/ml)		IL-10 (pg/ml)	
	Blood	Membrane	Blood	Membrane
Control	14.39±0.94	10.44±1.82	37.56±9.34	30.16±2.18
IBS-D	20.12±1.03^a^	15.97±0.83^a^	25.68±14.36^a^	21.56±1.73^a^

a P<0.05 compared with the control group. IL, interleukin; IBS, irritable bowel syndrome; IBS-D, diarrhea-predominant IBS.

**Table III.** Comparisons of IL-1β and IL-10 levels between the IBS groups.

IBS group	IL-1β (pg/ml)		IL-10 (pg/ml)	
	Blood	Membrane	Blood	Membrane
Non-anxiety-depression	18.35±1.37	16.28±0.97	30.34±7.33	28.45±3.34
Anxiety-depression	21.65±0.78^a^	20.86±1.34^a^	27.36±8.24^a^	24.31±5.66^a^

a P<0.05 compared with the non-anxiety-depression IBS-D group. IL, interleukin; IBS, irritable bowel syndrome; IBS-D, diarrhea-predominant IBS.

**Table IV.** Comparisons of IL-1β- and IL-10-positive cells between the IBS and control groups.

Group	IL-1β	IL-10
Control	0.091±0.036	0.159±0.138
IBS-D	0.298±0.312^a^	0.083±0.045^a^

a P<0.05 compared with the control group. IL, interleukin; IBS, irritable bowel syndrome; IBS-D, diarrhea predominant IBS.

**Table V.** Comparisons of the IL-1β- and IL-10-positive cells between the IBS groups.

IBS group	IL-1β	IL-10
Non-anxiety-depression	0.234±0.124	0.104±0.257
Anxiety-depression	0.310±0.223^a^	0.075±0.097^a^

a P<0.05 compared with the non-anxiety-depression group. IL, interleukin; IBS, irritable bowel syndrome.

## References

[1] Occhipinti K, Smith JW (2012). Irritable bowel syndrome: a review and update. Clin Colon Rectal Surg.

[2] Lackner JM, Gudleski GD, Dimuro J, Keefer L, Brenner DM (2013). Psychosocial predictors of self-reported fatigue in patients with moderate to severe irritable bowel syndrome. Behav Res Ther.

[3] Stasi C, Rosselli M, Bellini M, Laffi G, Milani S (2012). Altered neuro-endocrine-immune pathways in the irritable bowel syndrome: the top-down and the bottom-up model. J Gastroenterol.

[4] Gonsalkorale WM, Perrey C, Pravica V, Whorwell PJ, Hutchinson IV (2003). Interleukin 10 genotypes in irritable bowel syndrome: evidence for an inflammatory component. Gut.

[5] Chan J, Gonsalkorale W, Perrey M (2000). IL-10 and TGF-beta genotype in irritable bowel syndrome: evidence to support an inflammatory component?. Gastroenterology.

[6] Longstreth GF, Thompson WG, Chey WD, Houghton LA, Mearin F, Spiller RC (2006). Functional bowel disorders. Gastroenterology.

[7] Fukudo S (2007). Role of corticotrophin-releasing hormone in irritable bowel syndrome and intestinal inflammation. J Gastroenterol.

[8] Spiller R, Campbell E (2006). Post-infectious irritable bowel syndrome. Curr Opin Gastroenterol.

[9] Wouters MM, Boeckxstaens GE (2011). Neuroimmune mechanisms in functional bowel disorders. Neth J Med.

[10] Camilleri M (2009). Evolving concepts of the pathogenesis of irritable bowel syndrome: to treat the brain or the gut?. J Pediatr Gastroenterol Nutr.

[11] Sugaya N, Nomura S (2008). Relationship between cognitive appraisals of symptoms and negative mood for subtypes of irritable bowel syndrome. Biopsychosoc Med.

[12] Nicholl BI, Halder SL, Macfarlane GJ (2008). Psychosocial risk markers for new onset irritable bowel syndrome - results of a large prospective population based study. Pain.

[13] Camilleri M, Fealy RD (1990). Idiopathic autonomic denervation in eight patients presenting with functional gastrointestinal disease: A causal association?. Dig Dis Sci.

[14] Schiepers OJ, Wichers MC, Maes M (2005). Cytokines and major depression. Prog Neuropsychopharmacol Biol Psychiatry.

[15] Smulevich AB, Rappoport SI, Syrkin AL (2002). Visceral neuroses: clinical approaches to the problem. Zh Nevrol Psikhiatr Im S S Korsakova.

[16] Chadwick VS, Chen W, Shu D (2002). Activation of the mucosal immune system in irritable bowel syndrome. Gastroenterology.

[17] Li Y, Wang Y, Zuo X (2004). Visceral perception thresholds after rectal thermal and pressure stimuli in irritable bowel syndrome patients. J Gastroenterol Hepatol.

[18] Smart HL, Atkinson M (1987). Abnormal vagal function in irritable bowel syndrome. Lancet.

[19] Dinan TG, Quigley EM, Ahmed SM (2006). Hypothalamic-pituitary-gut axis dysregulation in irritable bowel syndrome: plasma cytokines as a potential biomarker?. Gastroenterology.

[20] Drossman DA (2006). The functional gastrointestinal disorders and the Rome III process. Gastroenterology.

[21] Heller F, Florian P, Bojarski C (2005). Interleukin-13 is the key effector Th2 cytokine in ulcerative colitis that affects epithelial tight junctions, apoptosis, and cell restitution. Gastroenterology.

[22] Liebregts T, Adam B, Bredack C (2007). Immune activation in patients with irritable bowel syndrome. Gastroenterology.

[23] Akbar A, Yiangou Y, Facer P, Walters JR, Anand P, Ghosh S (2008). Increased capsaicin receptor TRPV1-expressing sensory fibres in irritable bowel syndrome and their correlation with abdominal pain. Gut.

[24] Binimelis J, Webb SM, Monés J (1987). Somatostatin and the irritable bowel syndrome. Lancet.

